# 靶向CD52嵌合抗原受体T细胞的制备及其抗白血病作用研究

**DOI:** 10.3760/cma.j.issn.0253-2727.2022.04.003

**Published:** 2022-04

**Authors:** 琰 刘, 钰 刘, 克晶 唐, 兆琪 陈, 峻黎 牟, 颖茜 徐, 海燕 邢, 征 田, 青 饶, 敏 王, 建祥 王

**Affiliations:** 中国医学科学院北京协和医学院血液病医院（中国医学科学院血液学研究所），实验血液学国家重点实验室，国家血液系统疾病临床医学研究中心，细胞生态海河实验室，天津市血液病细胞治疗研究重点实验室，天津 300020 State Key Laboratory of Experimental Hematology, National Clinical Research Center for Blood Diseases, Haihe Laboratory of Cell Ecosystem, Institute of Hematology & Blood Diseases Hospital, Chinese Academy of Medical Sciences & Peking Union Medical College, Tianjin 300020, China

**Keywords:** CD52, 嵌合抗原受体, 免疫治疗, CD52, Chimeric antigen receptor T cells, Immunotherapy

## Abstract

**目的:**

构建一种靶向CD52的嵌合抗原受体T细胞（CD52 CAR-T），探索其对CD52^+^白血病的治疗效果。

**方法:**

应用分子克隆技术构建以CD52为抗原结合区的CD52 scFv、4-1BB为共刺激分子的二代CAR-T细胞。慢病毒载体包装后感染T细胞，制备CD52 CAR-T细胞，通过流式细胞术检测CD52 CAR-T细胞CD4、CD8细胞亚群及其分化状态。通过体外杀伤实验、脱颗粒实验及细胞因子的释放等功能实验，观察CD52 CAR-T细胞对CD52^+^白血病细胞的特异性细胞毒作用。

**结果:**

①成功构建了pCDH-CD52 scFv-CD8α-4-1BB-CD3ζ-GFP表达载体，感染人T细胞，获得靶向CD52的CAR-T细胞。②感染CD52 CAR表达载体的第6天，T细胞中表达CD52的细胞可被清除［CD52 CAR-T组CD52^+^ T细胞为（4.48±4.99）％，Vector-T组为（56.58±19.8）％，*P*＝0.011］。③CD52 CAR-T的自我杀伤不会影响T细胞的增殖，培养后期CD52 CAR-T组的增殖速度高于Vector-T组。④T细胞表型检测发现CD52 CAR可以促进CAR-T细胞向中心记忆T细胞及效应记忆T细胞分化，减少CAR-T细胞向终末效应细胞分化。⑤CD52 CAR-T细胞与HuT78-19t及MOLT4-19t靶细胞以效靶比1∶1共培养24 h，HuT78-19t细胞可被清除，而对MOLT4-19t细胞无明显杀伤作用［（2.66±1.60）％对（56.66％±5.74）％，*P*<0.001］。⑥脱颗粒实验显示，HuT78-19t细胞对CD52 CAR-T细胞的激活作用显著高于MOLT4-19t细胞［（57.34±11.25）％对（13.06±4.23）％，*P*<0.001］。⑦CD52 CAR-T与HuT78-19t细胞共培养48 h上清中细胞因子分泌水平［IFN-γ（3706±226）pg/ml、TNF-α（1732±560）pg/ml］远高于MOLT4-19t细胞组［IFN-γ（1577±846）pg/ml、TNF-α（74±12）pg/ml］（*P*值均<0.01）。

**结论:**

该研究成功制备了具有抗白血病作用的CD52 CAR-T细胞，为进一步的靶向CD52的免疫治疗奠定了基础。

## Abstract (en)

**Objective:**

To construct chimeric antigen receptor (CAR) T cells targeting CD52 (CD52 CAR-T) and validate the effect of CD52 CAR-T cells on CD52-positive leukemia.

**Methods:**

A second-generation CD52-targeting CAR bearing 4-1BB costimulatory domain was ligated into a lentiviral vector through molecular cloning. Lentivirus was prepared and packaged by 293 T cells with a four-plasmid system. Fluorescein was used to label cell surface antigens to evaluate the phenotype of CD52 CAR-T cells after infection. Flow cytometry and ELISA were used to evaluate the specific cytotoxicity of CD52 CAR-T cells to CD52-positive cell lines in vitro.

**Results:**

①A pCDH-CD52scFv-CD8α-4-1BB-CD3ζ-GFP expressing plasmid was successfully constructed and used to transduce T cells expressing a novel CD52-targeting CAR. ②On day 6, CD52-positive T cells were almost killed by CD52-targeted CAR-T post lentivirus transduction [CD52 CAR-T (4.48 ± 4.99)％, *vs* Vector-T (56.58±19.8)％, *P*＝0.011]. ③T cells transduced with the CAR targeting CD52 showed low levels of apoptosis and could be expanded long-term ex vivo. ④The CD52 CAR could promote T cell differentiation into central and effector memory T cells, whereas the proportion of T cells with a CD45RA^+^ effector memory phenotype were reduced. ⑤CD52 CAR-T cells could specifically kill CD52-positive HuT78-19t cells but had no killing effect on CD52-negative MOLT4-19t cells. For CD52 CAR-T cells, the percentage of residual of HuT78-19t cells was (2.66±1.60)％ at an the E:T ratio of 1∶1 for 24 h, while (56.66±5.74)％ of MOLT4-19t cells survived (*P*<0.001). ⑥The results of a degranulation experiment confirmed that HuT78-19t cells significantly activated CD52 CAR-T cells but not MOLT4-19t cells [(57.34±11.25)％ *vs* (13.06± 4.23)％, *P*<0.001]. ⑦CD52 CAR-T cells released more cytokines when co-cultured with HuT78-19t cells than that of vector-T cells [IFN-γ: (3706±226) pg/ml, *P*<0.001; TNF-α: (1732±560) pg/ml, *P*<0.01].

**Conclusion:**

We successfully prepared CD52 CAR-T cells with anti-leukemia effects, which might provide the foundation for further immunotherapy.

肿瘤过继细胞疗法（ACT）已经成为继放化疗等传统治疗方法后，治疗难治性造血系统恶性肿瘤的新一代的治疗策略[1]。其中，嵌合抗原受体T细胞（CAR-T细胞）作为其中一种免疫疗法，在血液肿瘤中已经被广泛应用，特别是以CD19作为靶抗原的CAR-T细胞免疫治疗，完全改变了复发难治性急性B淋巴细胞白血病（B-ALL）、非霍奇金淋巴瘤（NHL）的治疗格局[2]–[3]。但其他血液肿瘤，如慢性淋巴细胞白血病（CLL）、急性T淋巴细胞白血病（T-ALL）等患者仍面临着治疗反应率较低、疾病缓解期较短、预后较差的现状[4]–[6]。探索更多适合的治疗靶点，可进一步提高CAR-T细胞适用范围，使更多患者获益。

CD52分子是一种广泛表达的糖磷脂酰肌醇连接蛋白，属于短链糖基磷脂酰肌醇锚定糖蛋白家族[7]–[8]。CD52分子高表达于淋巴细胞，在造血干祖细胞上不表达[9]–[11]。很多恶性血液疾病中都能检测到CD52的表达，如CLL、NHL、T-ALL及FLT3-ITD突变的急性髓系白血病（AML）。阿仑单抗（Alemtuzumab）是一种完全人源化的CD52单克隆抗体，2001年已被FDA批准用于治疗难治复发性CLL[12]。因此，CD52是一个有开发潜能的肿瘤抗原靶点[13]–[15]。

本研究中，我们成功构建以CD52 scFv为抗原结合区的二代CAR结构，并制备CD52 CAR-T细胞，通过体外功能实验初步探讨其抗白血病作用。

## 材料与方法

一、主要材料及试剂

胎牛血清购自美国Biowest公司；CD3/CD28磁珠、DMEM培养基、RPMI 1640培养基购自美国Gibco公司；KBM581培养基购自美国Corning公司；重组人IL-2、IFN-γ和TNF-α ELISA试剂盒均购自美国R&D公司；pMD19-simple T载体购自北京擎科生物科技有限公司；小鼠抗体scFv基因扩增试剂盒购自江苏Public Protein/Plasmid Library公司；限制性内切酶NheⅠ和NotⅠ购自美国NEB公司；RossetteSep T细胞富集液购自美国Stem Cell公司；人淋巴细胞分离液购自天津市灏洋生物制品科技有限责任公司；重组人CD52-hFc融合蛋白购自美国Sino Biological公司；本研究所用抗体均购自美国Biolegend公司。

二、细胞培养

人急性淋巴母细胞白血病细胞系HuT78和MOLT4细胞均购自美国模式培养物集存库（ATCC），培养于含10％胎牛血清的RPMI 1640培养基中；293T细胞系购自ATCC，培养于含15％胎牛血清的DMEM培养基中；人T细胞培养于含5％胎牛血清和50 U/ml 重组人IL-2的KBM581培养基中。

三、pCDH-CD52 scFv-CD8α-4-1BB-CD3ζ CAR慢病毒载体的构建

提取本所院自主研发的鼠抗人CD52单克隆抗体杂交瘤（HI186）细胞株的总RNA，逆转录合成cDNA文库。使用小鼠抗体scFv基因扩增试剂盒经PCR扩增鼠抗人CD52单克隆抗体轻链可变区（VL）和重链可变区（VH）的基因片段。琼脂糖凝胶电泳分离并回收VL、VH片段。将VL、VH连接至pMD19-simple T载体并测序，根据测序结果确定CD52单克隆抗体VL、VH的核酸序列。应用重组方法构建VL-（G4S）_3_-VH的CD52 scFv片段：将CD52 scFv片段连接到实验室前期已构建的pCDH-CD8α-4-1BB-CD3ζ-GFP质粒中[16]–[17]，构建pCDH-CD52 scFv-CD8α-4-1BB-CD3ζ CAR-GFP表达载体质粒。将构建成功的表达载体用NheⅠ和NotⅠ进行酶切鉴定。pCDH-GFP表达载体质粒（Vector）为空白对照。

四、CD52 CAR-T细胞的制备

利用慢病毒四质粒包装系统转染293T细胞，收集24和48 h培养上清，应用超速离心沉淀法浓缩与纯化慢病毒。使用RossetteSep T细胞富集液和人淋巴细胞分离液从健康人群外周血中分离富集CD3^+^ T细胞，以CD3/CD28磁珠培养刺激24 h，感染CD52 CAR或者Vector慢病毒。感染第4天，用重组人CD52-hFc融合蛋白标记CAR-T细胞或Vector-T细胞，用APC抗人IgG FC抗体分别标记CD52 CAR-T细胞和Vector-T细胞，流式细胞术检测T细胞的感染效率。

五、CD52 CAR-T细胞对T细胞表型及生物学功能的影响

1. T细胞CD52表达情况：分别在T细胞感染后的第3、6天，用APC抗人CD52抗体分别标记CD52 CAR-T细胞和Vector-T细胞，流式细胞术检测T细胞CD52表达情况。

2. T细胞增殖情况：在T细胞培养的过程中，从第3天开始，每3天计数CD52 CAR-T细胞和Vector-T细胞活细胞数目，计算T细胞增殖情况。

3. CD4/CD8比例检测：在T细胞培养的第7天，选取PE/Cy7抗人CD4抗体和PerCP抗人CD8抗体分别标记CD52 CAR-T细胞和Vector-T细胞，流式细胞术检测CD4^+^和CD8^+^ CAR-T细胞比例。

4. T细胞分化状态检测：在T细胞培养的第7天，选取APC/Cy7抗人CD45RA抗体和PE抗人CCR7抗体分别标记CD52 CAR-T细胞和Vector-T细胞，流式细胞术分析两组不同亚型T细胞的比例，分析CAR-T细胞的分化状态。

六、CD52 CAR-T细胞体外抗白血病细胞作用

1. CD52^+^靶细胞的选择：选用CD52^+^白血病细胞系HuT78细胞株为阳性靶细胞，CD52^−^白血病细胞系MOLT4细胞株为阴性靶细胞，在此基础上以慢病毒感染截短型CD19表面蛋白，得到HuT78-19t和MOLT4-19t细胞。用APC抗人CD52抗体分别标记HuT78-19t和MOLT4-19t细胞，流式细胞术检测CD52的表达水平。

2. CD52 CAR-T细胞特异性杀伤功能检测：在T细胞培养的第7天，将CD52 CAR-T细胞或Vector-T细胞与HuT78-19t或MOLT4-19t细胞以效靶比1∶4、1∶2、1∶1、2∶1共培养24 h，用APC抗人CD19抗体标记HuT78-19t和MOLT4-19t细胞，流式细胞术检测靶细胞残留比例。

3. CD52 CAR-T细胞特异性激活检测：CD52 CAR-T细胞或Vector-T细胞与HuT78-19t或MOLT4-19t以效靶比1∶1共培养于含PE抗人CD107a抗体和人重组IL-2因子的培养体系中放置在37 °C孵箱中孵育5 h，流式细胞术检测CAR-T细胞中CD107a^+^细胞比例。

4. 细胞因子释放检测：取CD52 CAR-T细胞或Vector-T细胞与HuT78-19t或MOLT4-19t以1∶1的效靶比共培养48 h，取培养上清液，通过ELISA法检测上清液中TNF-α和IFN-γ的表达水平。

七、统计学处理

采用GraphPad Prism 8.0软件对本实验的所有数据进行处理及统计学分析。所有实验均重复至少3次，数据以均数±标准差表示，组间比较采用双尾*t*检验。*P*<0.05为差异有统计学意义。

## 结果

1. pCDH-CD52 scFv-CD8α-4-1BB-CD3ζ CAR表达载体的构建：从小鼠抗人CD52单克隆抗体（HI186）杂交瘤获得VL和VH序列，按照VL在前VH在后的顺序用（G4S）_3_的linker连接为能特异性识别CD52的抗原识别区CD52 scFv，连接到实验室前期构建的含有pCDH-CD8α-4-1BB-CD3ζ-GFP片段的质粒，成功构建了靶向CD52的二代CAR的慢病毒表达载体（图1A）。使用限制性内切酶双酶切鉴定，表明阳性克隆含有目的条带（1411 bp）和载体片段（7303 bp）（图1B），测序鉴定序列正确。

**图1:**
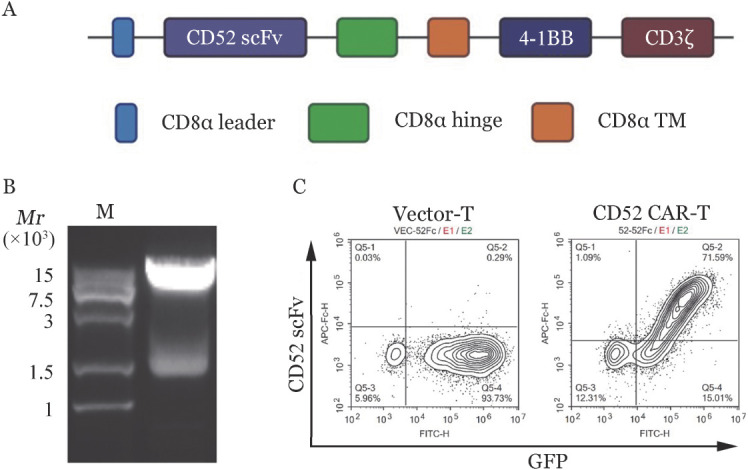
pCDH-CD52 scFv-CD8α-4-1BB-CD3ζCAR表达载体的构建 A：CD52 scFv-CD8α-4-1BB-CD3ζ CAR载体结构示意图；B：慢病毒载体进行酶切鉴定琼脂糖凝胶电泳图；C：流式细胞术检测Vector-T和CD52 CAR-T细胞感染效率。CAR：嵌合抗原受体；M：Marker

2. CD52 CAR-T细胞的制备：通过慢病毒载体包装、感染T细胞获得CD52 CAR-T及Vector-T细胞，使用重组人CD52-hFc融合蛋白标记T细胞，通过T细胞表面CD52 CAR的表达和GFP荧光检测T细胞的感染效率，由图1C可见，CD52 CAR-T组感染效率在70％左右，Vector-T组感染效率在90％以上。

3. CD52 CAR-T细胞CD52表达和T细胞增殖情况：慢病毒感染人T细胞第3天，CD52 CAR-T细胞的CD52表达与Vector-T细胞无明显差异［CAR-T：（15.91±8.41）％；Vector-T：（20.35±12.1）％］。培养至第6天，Vector-T细胞CD52表达为（56.58±19.8）％，CAR-T细胞CD52表达仅为（4.48±4.99）％（*P*＝0.011），提示CD52 CAR-T细胞能识别本身CD52分子并激活T细胞清除CD52^+^ T细胞（图2A）。

**图2:**
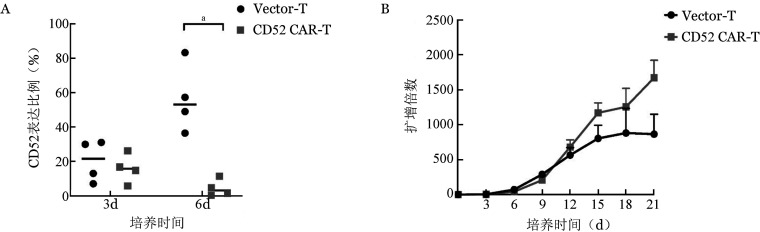
流式细胞术检测Vector-T和CD52 CAR-T细胞中CD52表达变化（A）及细胞增殖情况（B）（实验重复4次，^a^*P*<0.05）

从慢病毒感染T细胞后第3天开始，每3天计数CD52 CAR-T细胞和Vector-T细胞活细胞数目，计算两组T细胞增殖情况。由图2B可看出，CD52 CAR-T细胞的自我杀伤未影响T细胞增殖，CD52 CAR-T细胞可大量扩增。培养至第12天时，CD52 CAR-T细胞增殖倍数已超过Vector-T细胞，培养中后期CD52 CAR-T细胞的增殖快于Vector-T细胞。

4. CAR对T细胞亚群的影响：通过PE/Cy7抗人CD4抗体和PerCP抗人CD8抗体标记感染后第7天的CD52 CAR-T细胞和Vector-T细胞，检测CD4、CD8亚群的分布。由图3A可看出，CD52 CAR-T细胞和Vector-T细胞中CD4^+^ T细胞比例分别为（37.6±13.9）％和（31.29±10.02）％（*P*＝0.43），CD52 CAR-T细胞和Vector-T细胞中CD8^+^ T细胞比例分别为（57.09±15.81）％和（62.33±12.78）％（*P*＝0.58）。两组在CD4和CD8亚群比例上差异无统计学意义。

**图3:**
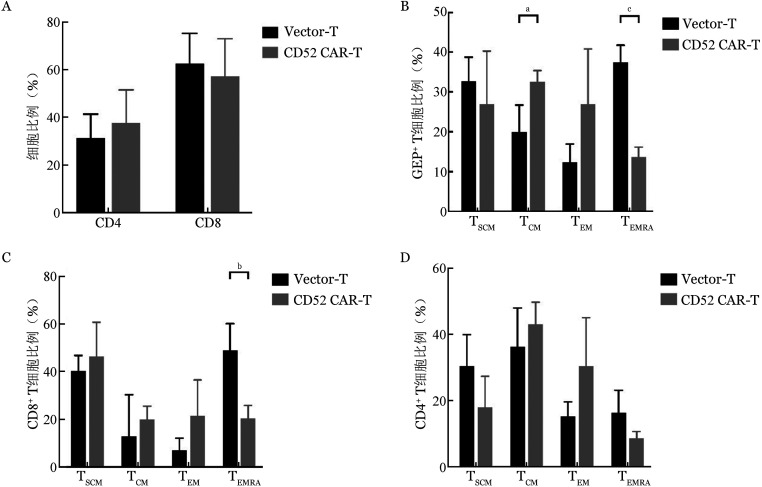
流式细胞术检测Vector-T和CD52 CAR-T细胞中CD4^+^、CD8^+^ T细胞比例（A）及细胞分化状态（B～D）（实验重复5次，^a^*P*<0.01、^b^*P*<0.001、^c^*P*<0.0001）

通过APC/Cy7抗人CD45RA抗体和PE抗人CCR7抗体标记感染后第7天的CD52 CAR-T细胞和Vector-T细胞，检测CD52 CAR对CAR-T细胞、CD8^+^ CAR-T细胞和CD4^+^ CAR-T细胞分化状态的影响。与Vector-T细胞相比，CD52 CAR-T细胞中心记忆T细胞（T_CM_）亚群、效应记忆T细胞（T_EM_）亚群比例均升高（*P*＝0.005；*P*＝0.056）, 终末分化效应细胞（T_EMRA_）亚群比例明显降低（*P*<0.0001）。T_CM_和T_EM_比例的增加在CD8^+^ CAR-T细胞和CD4^+^ CAR-T细胞中都有相同的趋势，提示CD52 CAR可诱导T细胞优先向T_CM_和T_EM_分化，减少向T_EMRA_分化（图3B～D）。

5. CD52 CAR-T对CD52^+^白血病细胞的特异性杀伤作用：应用流式细胞术检测靶细胞HuT78-19t和MOLT4-19t细胞系CD52表达阳性率，分别为90.44％和0.19％（图4A）。HuT78-19t和MOLT4-19t细胞系表达CD52的平均荧光强度（MFI）分别为12 823和2 071（图4B）。

**图4:**
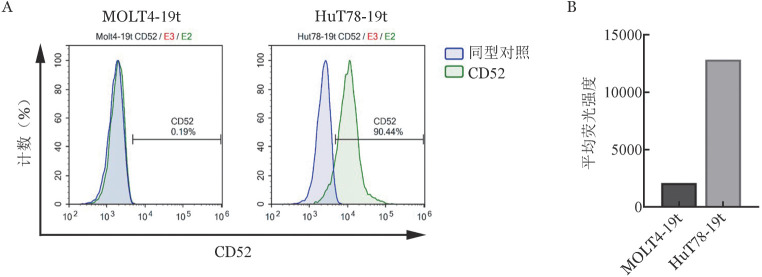
流式细胞术检测白血病细胞系CD52阳性细胞比例（A）及平均荧光强度（B）

将效应细胞与靶细胞以1∶4、1∶2、1∶1、2∶1四种效靶比铺板共培养24 h，CD52 CAR-T组各效靶比组残留HuT78-19t细胞比例分别为（57.15±1.69）％、（19.07±7.02）％、（2.66±1.60）％和（0.26±0.18）％，Vector-T组残留HuT78-19t细胞比例分别为（84.37％±4.89）％、（73.92％±5.78）％、（56.66％±5.74）％和（36.64％±6.43）％。上述结果显示，当效靶比为1∶1时，CD52 CAR-T细胞24 h几乎完全清除HuT78-19t细胞（*P*<0.001）（图5A）。CD52 CAR-T细胞和Vector-T细胞与阴性靶细胞MOLT4-19t共培养的体系中，两组MOLT4-19t细胞比例无明显差异（图5B）。

**图5:**
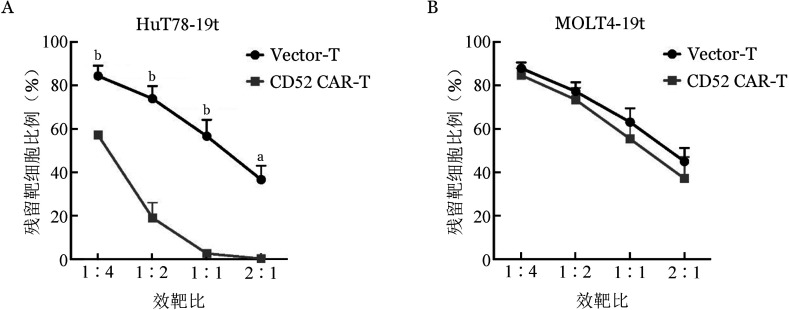
流式细胞术检测Vector-T及CD52 CAR-T细胞与靶细胞HuT78-19t（A）Molt4-19t（B）共培养24 h残留的靶细胞比例（实验重复5次，^a^*P*<0.001、^b^*P*<0.0001）

脱颗粒实验证实，CD52 CAR-T细胞可以被CD52^+^靶细胞迅速有效激活，CD52 CAR-T细胞与HuT78-19t细胞共培养5 h后，细胞激活率为（57.34±11.25）％，显著高于与MOLT4-19t细胞共培养的激活率［（13.06±4.23）％，*P*<0.0001］，而Vector-T与HuT78-19t和MOLT4-19t两种靶细胞共培养，细胞激活率仅为（2.94±0.70）％、（0.67±0.23）％（图6A）。

用ELISA法检测CD52 CAR-T细胞与白血病细胞以1∶1的效靶比共培养48 h后培养上清中细胞因子IFN-γ、TNF-α的水平。结果如图6B、6C所示，CD52^+^的HuT78-19t与CAR-T共培养上清中IFN-γ、TNF-α细胞因子水平为（3706±226）pg/ml及（1732±560）pg/ml，均较Vector-T组有显著性升高（*P*<0.001，*P*<0.01），在与CD52^−^的MOLT4-19t细胞的共培养上清中CAR-T分泌的IFN-γ、TNF-α水平显著降低，分别为（1577±846）pg/ml及（74±12）pg/ml。

**图6:**
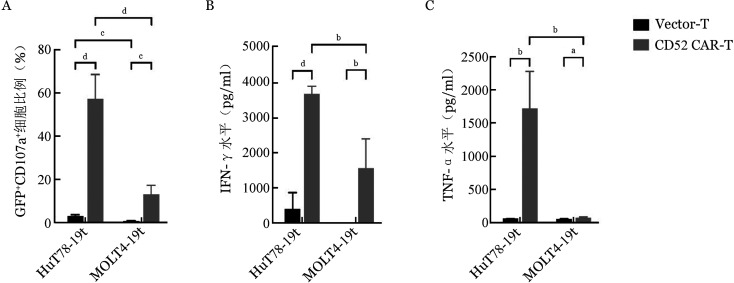
流式细胞术检测Vector-T、CD52 CAR-T细胞和靶细胞共培养CD107a^+^表达（A），ELISA法检测Vector-T、CD52 CAR-T细胞和靶细胞共培养48小时培养上清细胞细胞因子IFN-γ（B）和TNF-α（C）释放水平（*n*＝4，^a^ *P*<0.05、^b^ *P*<0.01、^c^ *P*<0.001、^d^ *P*<0.0001）

## 讨论

CAR-T细胞在血液肿瘤中已经被广泛应用，它可以不依赖主要组织相容性复合体（MHC）限制性直接识别肿瘤抗原，靶向并消除肿瘤细胞。CAR-T细胞疗法的出现改变了复发难治性B细胞白血病/淋巴瘤的治疗局面，特别是CD19作为靶抗原的CAR-T细胞免疫治疗已获得突破性进展，将成人B-ALL的完全缓解率提高到了80％以上[18]。随着CAR-T治疗临床需求的增加，利用肿瘤抗原多样化的特点，开发更多的抗原识别区，提供更多有效的治疗靶点是扩大CAR-T适用范围的主要途径。

CD52抗原又称CAMPATH-1抗原，是一种由12个氨基酸多肽组成的糖磷脂酰肌醇连接蛋白，属于短链糖基磷脂酰肌醇锚定糖蛋白家族[8]。CD52分子是一种分布比较广泛的抗原，在细胞表面成簇有序地排列，几乎所有的造血细胞都以不同的水平表达，其中T淋巴细胞和B淋巴细胞中表达最高，红细胞、血小板、精原细胞表面以及造血干祖细胞不表达CD52[9]–[11],[19]。目前研究表明，CD52是参与T细胞激活的抗原，且是诱导CD4^+^ T细胞生成调节性T细胞（Treg）的共刺激分子[20]–[21]。阿仑单抗是一种完全人源化的CD52单克隆抗体，2001年被FDA批准用于治疗难治复发型CLL[12]。除此之外，CD52在AML、T-ALL、NHL中均有表达。目前研究证实阿仑单抗在消除T-ALL患者微小残留病方面有效，但不良反应可能会限制阿仑单抗的进一步开发[22]。

本研究通过本所院自主研制的小鼠抗人CD52单克隆抗体杂交瘤细胞株（HI186），获得与阿仑单抗抗原识别区不同的CD52 scFv，该杂交瘤生产的单克隆抗体已成功商品化，可用于CD52表达的检测。抗原识别区是CAR-T细胞特异性识别肿瘤细胞的核心部分，相同的抗原，因其识别表位不同所表现出的亲和性不同，从而产生不同的治疗效果[23]–[24]。阿仑单抗所识别的是近膜端的氨基酸残基及部分GPI锚定位点，而HI186所识别的是远膜端连接于3位天冬酰胺的糖基[25]–[26]。

基于以上研究背景，我们成功构建了与阿仑单抗不同抗原识别区的嵌合抗原受体，获得CD52 CAR-T细胞，感染后第6天，CD52 CAR-T细胞本身CD52表达已通过自我杀伤被清除，且并未影响CAR-T细胞的扩增。通过对CD52 CAR-T细胞的表型检测，我们发现CD52 CAR-T可以促进CAR-T细胞向T_CM_和T_EM_分化，抑制CAR-T细胞向T_EMRA_分化，有利于提高T细胞的持久性。体外实验证实CD52 CAR-T可以被CD52^+^肿瘤细胞特异性激活，启动脱颗粒过程，同时分泌IFN-γ、TNF-α等Th1类细胞因子特异性的杀伤肿瘤细胞，同时，对CD52^−^肿瘤细胞没有脱靶效应。在培养过程中，我们发现培养后期CD52 CAR-T细胞本身CD52表达会逐渐上升，为解决这个的问题，我们采用CRISPR/Cas9技术，清除T细胞表面的CD52，构建不表达CD52的CAR-T细胞。目前，我们已证实敲除CD52后T细胞本身CD52表达不会随培养时间延长而升高，后续我们还会对敲除体系进行优化，并检测不表达CD52的CAR-T细胞的表型与功能。

综上所述，本研究成功构建的靶向CD52 抗原的第二代CAR-T细胞具有特异性的抗肿瘤作用，同时没有表现出脱靶效应。研究结果提示CD52是治疗CD52^+^肿瘤的有效靶点，CAR-T可以作为化疗后衔接骨髓移植的一个桥梁，为肿瘤患者个体化治疗提供新的选择，也为精准医学的发展提供新思路。
